# Effect of Heme Oxygenase-1 on Mitofusin-1 protein in LPS-induced ALI/ARDS in rats

**DOI:** 10.1038/srep36530

**Published:** 2016-11-10

**Authors:** Jianbo Yu, Ying Wang, Zhen Li, Shuan Dong, Dan Wang, Lirong Gong, Jia Shi, Yuan Zhang, Daquan Liu, Rui Mu

**Affiliations:** 1 Department of Anesthesiology, Tianjin Nan Kai Hospital, Tianjin Medical University, Tianjin, 300100, China; 2 Institute of Acute Abdominal Diseases of Integrated Traditional Chinese and Western Medicine, Tianjin, 300100, China

## Abstract

Acute lung injury (ALI)/acute respiratory distress syndrome (ARDS) is a common and important oxidative stress in the lung. Mitochondrial fusion responds to the normal morphology and function of cells and is finely regulated by mitochondrial fusion proteins, such as mitofusin-1 protein (Mfn1), mitofusin-2 protein (Mfn2) and optical atrophy 1 (OPA1). Additionally, Mfn1 has been identified as the most important protein in mitochondrial fusion. Heme oxygenase-1 (HO-1) is a stress-inducible protein that plays a critical role in protecting against oxidative stress. However, whether the protection of HO-1 is related to mitochondrial fusion is still a question. Thus, our *in vitro* and *in vivo* experiments aimed to identify the relationship between HO-1 and Mfn1. Here, we used Hemin and ZnPP-IX as treatments in an *in vivo* experiment. Then, HO-1 and Mfn1 were measured using RT-PCR and Western blotting. Supernatants were analyzed for MDA, SOD, and ROS. Our results implied that HO-1 upregulation suppressed oxidative stress induced by LPS, and the possible mechanism could be associated with Mfn1 and the PI3K/Akt pathway.

As is known, acute lung injury (ALI) and its more severe form, acute respiratory distress syndrome (ARDS), are common and important oxidative stress diseases that are caused by various factors, such as sepsis, trauma, and ischemia/reperfusion injury^1^. ALI/ARDS is characterized by a disruption of the endothelium and alveolar injury, resulting in an uncontrolled inflammatory response, including increasing release of reactive oxygen species (ROS), inflammatory cytokines, protein content and neutrophil accumulation^2^. Despite numerous studies that have been performed in recent years, the underlying mechanisms of ALI/ARDS are still unclear, resulting in a high mortality rate of 30% to 40%^3^. Concomitantly, previous studies identified an important mechanism leading to ALI/ARDS: the accumulation of ROS^4^ in cells, which can cause tissue damage, cell dysfunction and uncontrolled inflammatory responses. Its molecular mechanism might be related to the activation of inflammatory signaling, including the mitogen-activated protein kinases (MAPKs) and the NF-κB complex^5^.

Heme oxygenase-1 (HO-1), which is expressed in lung endothelial and epithelial cells, monocytes and neutrophils, is a stress-inducible protein that catalyzes the breakdown of heme to free iron, carbon monoxide and biliverdin in mammalian cells and acts as the rate-limiting step of heme degradation^6^. Additionally, it also works as a strong negative regulator in the development of oxidative stress, which is upregulated by many signaling pathways^7^, and its protection has been confirmed in myocardial ischemia-reperfusion injury, hepatic injury, brain hemorrhage, acute kidney injury and viral diarrhea^8^. Our previous findings showed strong evidence that HO-1 upregulation plays a pivotal role in the defense against oxidative stress and acts as a critical mediator of cellular homeostasis. Activating HO-1 with Hemin (a selective activator) and Znpp-IX (a potent inhibitor) can strikingly attenuate acute lung injury in rabbits and acute kidney injury in rats^9^,^10^. Furthermore, the PI3K/Akt pathway, which coordinates a variety of intracellular signals, has been clarified in many models for its protective effects such as enhancing HO-1 through the Nrf2/ARE axis and activing GSK kinase. If the HO-1 response to oxidative stress is too weak, cellular damage continues and apoptosis is initiated, and further tissue impairments can occur.

Mitochondria are double-membrane-bound subcellular organelles that are involved in various activities in mammalian cells, such as apoptosis, metabolism, tumor formation and oxidative stress^11^,^12^,^13^,^14^. Mitochondria fusion, stimulated by an energy shortage and oxidative stress, helps relieve stress by sharing the contents of damaged mitochondria as a form of compensation, contributing to the maintenance of ATP production and removing ROS derived from the mitochondria. Mitochondria undergo continuous fusion in response to various oxidative stresses, helping cells to adapt to challenges from the environment^15^. Recent studies have suggested that the key regulatory proteins of mitochondrial fusion are Mfn1, Mfn2 and OPA1^16^. Mfn1 and Mfn2 are located in the outer mitochondrial membrane, and OPA1 is located in the inner mitochondrial membrane^17^. Furthermore, Mfn1 plays a more crucial role than Mfn2 and OPA1 in regulating mitochondrial fusion, and it is essential for embryonic development^18^.

However, although studies have revealed the protective effects of HO-1 and mitochondrial fusion in attenuating oxidative stress, there is no answer to the question of whether the protective role of HO-1 is related to mitochondrial fusion and how HO-1 affects mitochondrial protein expression. To resolve this issue, we sought to create *in vitro* and *in vivo* models using alveolar macrophages and LPS-induced ALI/ARDS in rats. This study aimed to investigate the link between HO-1 and the mitochondrial fusion protein Mfn1. Subsequently, we attempted to determine the relevant specific signaling pathways. Importantly, our study findings contribute greatly to the understanding of the mechanisms of HO-1′s protective role and provide a new treatment strategy for AlI/ARDS.

## Results

### *In vivo* study

#### MDA contents and SOD activity

**Figure 1:**
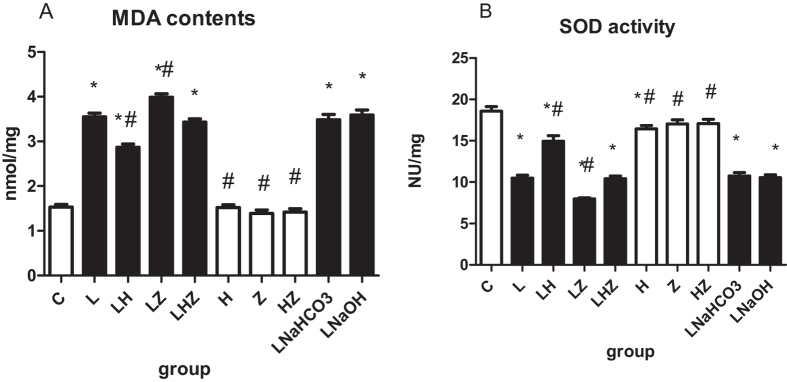
The comparisons of MDA contents and SOD activity among the 10 groups are shown in (**A**,**B**). (**A**) Shows the level of MDA contents. (**B**) Shows the level of SOD activity. Compared with control group(C). the MDA content in LPS group(L), LPS + NaHCO3 group(LNaHCO3) and LPS + NaOH group (LNaOH) was increased and SOD activity was decreased after the administration of LPS to rats (P < 0.05). Compared with LPS group(L). lower MDA content and higher SOD activity in LPS + Hemin group(LH) were observed (P < 0.05). After the application of ZnPP-IX, the MDA content in LPS + ZnPP-IX group(LZ) was upregulated and SOD activity was downregulated compared with those of LPS group (L) (P < 0.05). *p < 0.05 versus control group (C); ^#^*p* < 0.05 versus LPS group(L).

Consistent with the histology results, the comparisons of MDA contents and SOD activity among the 10 groups are shown in Fig. 1(A,B). The results clearly indicated that in LPS group(L), LPS + NaHCO3 group(LNaHCO3) and LPS + NaOH group (LNaOH), the administration of LPS to rats could induce oxidative stress, as reflected by an increased MDA content and decreased SOD activity compared with those of control group(C) (P < 0.05). Our findings also suggested that Hemin could reduce injury induced by LPS, as shown by the lower MDA content and higher SOD activity in LPS + Hemin group(LH) compared with those of LPS group(L)(P < 0.05). In addition, after the administration of Znpp-IX in LPS + ZnPP-IX group(LZ), the results indicated that MDA content was upregulated, whereas SOD activity was downregulated compared with LPS group(L)(P < 0.05). Interestingly, after the intervention of both Hemin and Znpp-IX, the results showed that the levels of MDA content and SOD activity were similar to those of LPS group(L) (P < 0.05).

#### HE Staining

**Figure 2:**
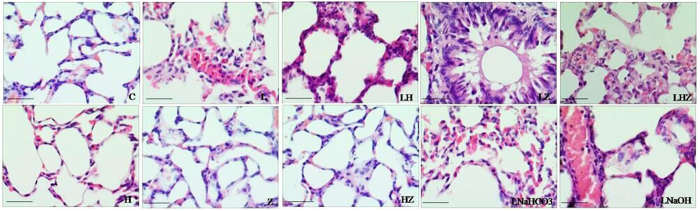
HE staining results in the lung tissue of rats(×400). Scale bars: 50 μm.In control group(C), no evident histological changes were observed. In LPS group(L), LPS + NaHCO3 group(LNaHCO3) and LPS + NaOH group (LNaOH), there were notable inflammatory cell infiltration, interalveolar septal thickening and hemorrhaging. In LPS + Hemin group(LH), the pathological injury in the lung tissues was ameliorated. By contrast, in LPS + ZnPP-IX group(LZ), the histological injury in lung tissues became more severe. However, in Hemin group(H), ZnPP-IX group(Z) and Hemin + ZnPP-IX group(HZ), the pathological changes showed no evident differences from those in control group(C).

To assess the pathological changes, HE staining was used in our study, as shown in Fig. 2 (×400). We found that rats in control group(C) showed no evident histological changes. Histological evaluation of LPS-exposed ALI revealed evidence of notable inflammatory cell infiltration, interalveolar septal thickening and hemorrhaging in LPS group(L), LPS + NaHCO3 group(LNaHCO3) and LPS + NaOH group (LNaOH). In LPS + Hemin group(LH), after Hemin treatment, the pathological injury in lung tissues was reversed. By contrast, in LPS + ZnPP-IX group(LZ), after the application of Znpp-IX, the histological injury in lung tissues became more severe. However, in Hemin group(H), ZnPP-IX group(Z) and Hemin + ZnPP-IX group(HZ), the pathological changes showed no evident differences from control group(C).

#### Real–time reverse transcriptase-polymerase chain reaction (RT-PCR) and Western blotting

**Figure 3:**
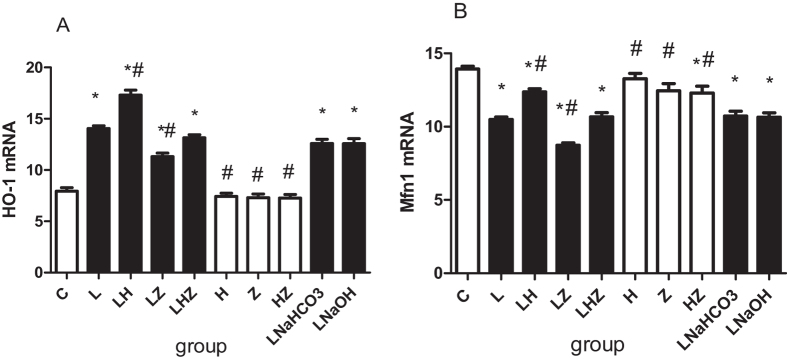
The *in vivo* mRNA expression of HO-1 and Mfn1 by RT-PCR. (**A**) Shows mRNA expression of HO-1. (**B**) Shows mRNA expression of Mfn1. Compared with control group(C), expression of Mfn1 was decreased, but expression of HO-1 was increased in LPS group(L), LPS + NaHCO3 group(LNaHCO3) and LPS + NaOH group (LNaOH)(P < 0.05). Compared with LPS group(L), mRNA expression of Mfn1 was significantly increased, and HO-1 expression was increased in LPS + Hemin group(LH) (P < 0.05). However, compared with LPS group(L), mRNA expression of Mfn1 and HO-1 were decreased in LPS + ZnPP-IX group(LZ) (P < 0.05). After using both Hemin and Znpp-IX, mRNA expression of Mfn1 in LPS + Hemin + ZnPP-IX group(LHZ) was similar to LPS group(L) (P < 0.05). *p < 0.05 versus control group(C); ^#^*p* < 0.05 versus LPS group(L).

**Figure 4:**
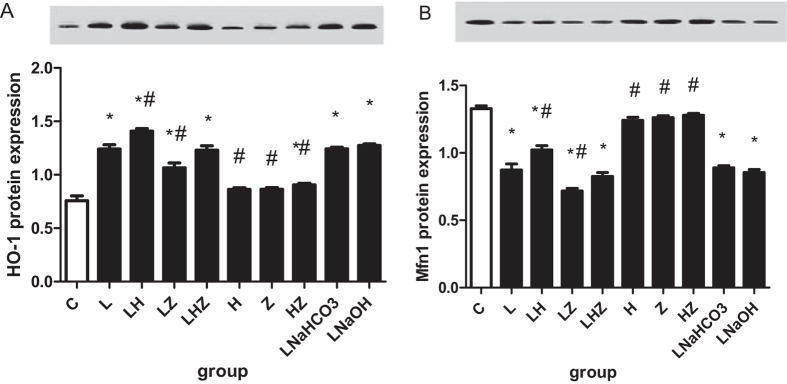
Western blot analysis of HO-1 and Mfn1 in the *in vivo* experiment. (**A**) Shows the protein expression results and the gel image for HO-1. (**B**) Shows the protein expression and the gel image for Mfn1. The gels were run under the same experimental conditions. Compared with control group(C), protein expression of Mfn1 was decreased, but expression of HO-1 was increased in LPS group(L), LPS + NaHCO3 group(LNaHCO3) and LPS + NaOH group(LNaOH)(P < 0.05). Compared with LPS group(L), protein expression of Mfn1 was significantly enhanced in LPS + Hemin group(LH) and was accompanied by increased HO-1 expression (P < 0.05). However, compared with LPS group(L), protein expression of Mfn1 was suppressed and accompanied by decreased HO-1 expression in LPS + ZnPP-IX group(LZ) (P < 0.05). Protein expression of Mfn1 in LPS + Hemin + ZnPP-IX group(LHZ) was similar to that of LPS group(L) (P < 0.05). *p < 0.05 versus control group(C); ^#^*p* < 0.05 versus LPS group(L).

To explore the mRNA and protein expression of HO-1 and Mfn1 *in vivo*, we performed RT-PCR and Western blot analysis (Figs 3 and 4). Compared with control group(C), the results revealed that in rats, exposure to LPS reduced mRNA and protein expression of Mfn1 but upregulated HO-1 expression in LPS group(L). A similar effect was observed in LPS + NaHCO3 group(LNaHCO3) and LPS + NaOH group (LNaOH)(P < 0.05). Compared with that of LPS group(L), mRNA and protein expression of Mfn1 was significantly enhanced in LPS + Hemin group(LH) after Hemin treatment, and HO-1 expression was also increased (P < 0.05). However, after the application of Znpp-IX, mRNA and protein expression of Mfn1 was suppressed in LPS + ZnPP-IX group(LZ), and HO-1 expression was decreased (P < 0.05). In group LPS + Hemin + ZnPP-IX group(LHZ), we used both Hemin and Znpp-IX; the results showed that mRNA and protein expression of Mfn1 was nearly the same as that in LPS group(L) and similar to that in control group(C) (P < 0.05).

### *In vitro* study

#### ROS, MDA contents and SOD activity exposure to LPS conditions

In the control group, the levels of ROS and MDA production were maintained at low concentrations, whereas SOD activity was maintained at a high concentration (P < 0.05, Table 1). After LPS treatment, the levels of ROS and MDA contents were dramatically increased with decreased SOD activity in the LPS group(L) compared with the control group(C) (P < 0.05, Table 1). However, after Hemin pretreatment, in the LPS + Hemin group(LH), our data showed that the levels of ROS and MDA contents were downregulated compared with those of the LPS group(L). Concomitantly, SOD activity was upregulated (P < 0.05, Table 1). Moreover, we examined the levels of ROS and the MDA contents and the SOD activity in the LPS + ZnPP-IX group(LZ). Our results suggested that treatment with ZnPP-IX increased the levels of ROS and MDA contents, whereas it decreased the levels of SOD activity compared with those of the LPS group(L) (P < 0.05, Table 1).

#### HO-1 and Mfn1 expression and the PI3K/AKT pathway in macrophages

**Figure 5:**
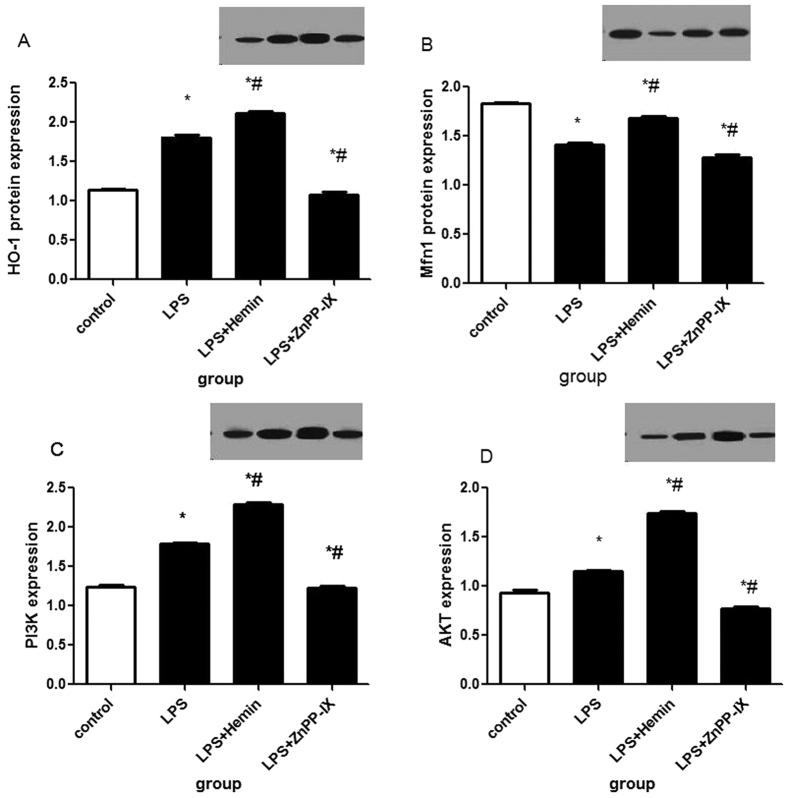
Western blot analysis in the *in vitro* study. In the *in vitro* study, we treated cells with 20 μM Hemin and (or) 10 μM ZnPP for 1 h prior to LPS treatment and continued the incubation for 24 h. As illustrated in (**A**–**D**), HO-1 expression was upregulated in the cells with LPS and pretreatment, compared with that of the unstimulated cells in the control group(C) (P < 0.05; (**A**). To investigate whether HO-1 impacts Mfn1 in LPS-induced rat alveolar macrophages, we analyzed the expression of the mitochondrial fusion protein Mfn1. As our results indicated, LPS significantly decreased Mfn1 expression compared with that of the control group(C) (P < 0.05); however, after Hemin pretreatment, Mfn1 expression was upregulated compared with that of the LPS group(L) (P < 0.05; (**B**). In addition, it has been reported that HO-1 is regulated by the PI3K/Akt pathway, and we thus investigated PI3K/Akt expression in LPS-induced rat alveolar macrophages. As our data showed, similar to HO-1 expression, PI3K/Akt expression was remarkably enhanced in the LPS group(L) compared with that of the control group(C) (P < 0.05; (**C**,**D**). Moreover, in the LPS + Hemin group(LH), the level of PI3K/Akt expression was upregulated to a higher degree compared with that of the LPS group(L) (P < 0.05; (**C**,**D**). By contrast, after ZnPP-IX pretreatment, our data showed a lower level of PI3K/Akt expression in the LPS + ZnPP-IX group(LZ) compared to that of the LPS group(L) (P < 0.05; (**C**,**D**).

In the *in vitro* study, we treated cells with 20 μM of Hemin and (or) 10 μM of ZnPP for 1 h prior to LPS treatment and continued the incubation for 24 h. As illustrated in Fig. 5(A–D), HO-1 expression was upregulated in the cells with LPS and pretreatment compared with that of the unstimulated cells in the control group(C) (P < 0.05; Fig. 5A). To investigate whether HO-1 impacts Mfn1 in LPS-induced rat alveolar macrophages, we analyzed the expression of the mitochondrial fusion protein Mfn1. As our results indicated, LPS significantly decreased Mfn1 expression compared with that of the control group(C) (P < 0.05); however, after Hemin pretreatment, Mfn1 expression was upregulated compared with that of the LPS group(L) (P < 0.05; Fig. 5B). In addition, it has been reported that HO-1 is regulated by the PI3K/Akt pathway, and we thus investigated PI3K/Akt expression in LPS-induced rat alveolar macrophages. As our data show, similar to HO-1 expression, PI3K/Akt expression was remarkably enhanced in the LPS group(L) compared with that of the control group(C) (P < 0.05; Fig. 5C,D). Moreover, in the LPS + Hemin group(LH), the level of PI3K/Akt expression was upregulated to a higher degree compared with that of the LPS group(L)(P < 0.05; Fig. 5C,D). By contrast, after ZnPP-IX pretreatment, our data showed a lower level of PI3K/Akt expression in the LPS + ZnPP-IX group(LZ) compared with that of the LPS group(L) (P < 0.05; Fig. 5C,D).

## Discussion

To our knowledge, the most common indirect pulmonary injury leading to ALI/ARDS is caused by LPS from the outer cell wall of Gram-negative bacteria^19^. Therefore, our *in vivo* study used international studies and our previous studies to select the intravenous LPS 5 mg/kg method to model ALI/ARDS in rats^20^. Consistent with earlier findings, after an injection of LPS for 6 h, lung inflammatory cells were increased, and IL-6 and IL-10 levels were significantly increased; therefore, we used 6 h as the observation point for our study^21^. Moreover, to determine the role of HO-1 induction in LPS-induced ALI/ARDS, we also treated with ZnPP-IX, which acts as a selective competitive enzyme for HO-1 compared to other microsomal enzymes^22^. NaHCO3 and NaOH are solvents for Znpp-IX and Hemin. Our results suggested that solvents, Hemin or Znpp-IX seem to have no effect on LPS-induced ALI in rats. SOD is one of the important antioxidant enzymes that removes ROS, and cell SOD activity can reflect the body’s ability to remove oxygen free radicals^23^. MDA is the product that oxygen free radicals react with in biological membrane unsaturated fatty acids. Its content can reflect the degree of lipid peroxidation, indirectly reflecting cell damage^24^. Therefore, the levels of ROS, SOD activity and MDA contents were measured to reflect the extent of oxidative stress.

As mentioned above, HO-1 has an effective function to reduce oxidative stress, and it is an important antioxidant enzyme^25^,^26^. The protective mechanisms of HO-1 might be related to its byproducts biliverdin and carbon monoxide, and it releases iron, which are strong antioxidants in mammals^27^. The induction of HO-1 occurs as an adaptive and beneficial response in many varied tissues and cellular injury models including sepsis, ischemia reperfusion, hyperoxia, hypoxia, and other oxidative stress^28^. Our previous studies also showed that the induction of HO-1 by LPS has a protective effect on the lung, kidney and liver^8^,^9^,^28^. Recently, studies have shown that the mechanism is likely regulated via the NF-κB and Nrf2/HO-1 signaling pathway^29^.

In this study, our results suggested that after LPS pretreatment in rats, we established a model of ALI/ARDS *in vivo*, as obvious injury could be observed in the pathological changes of the lung tissues. Meanwhile, oxidative stress occurred, as decreased SOD activity and enhanced MDA contents were detected. Consistent with previous studies, HO-1 was upregulated as a response to LPS-induced oxidative stress. Moreover, after Hemin treatment (a selective activator of HO-1), not only was higher HO-1 expression was detected, but oxidative stress injury was relieved, indicating that, indeed, HO-1 had a protective effect in the defense against oxidative stress. By contrast, after Znpp-IX treatment (a selective inhibitor of HO-1), the results were reversed. In the *in vitro* experiment, after Hemin pretreatment in rat alveolar macrophages, our findings also suggested that HO-1 expression was dramatically increased. At the same time, oxidative stress was decreased, as lower ROS and MDA content and higher SOD activity were detected. However, after ZnPP-IX pretreatment, the oxidative stress was more severe, which indicated the protective effect of HO-1.

Furthermore, in our study, we found that the level of Mfn1 expression was downregulated and was accompanied by elevated HO-1 expression in both LPS-induced ALI/ARDS rats and in rat alveolar macrophages. Interestingly, after Hemin treatment, Mfn1 expression was upregulated. By contrast, after we administered Znpp-IX, Mfn1 expression was downregulated. This finding led to speculation that HO-1 could affect the mitochondrial fusion protein Mfn1 expression to relieve oxidative stress induced by LPS. Furthermore, to determine the specific mechanism of how HO-1 affected Mfn1, we focused on the PI3K/Akt pathway, as previous studies have demonstrated that its activation was related to the upregulation of HO-1. Our data suggested that in addition to HO-1 upregulation, PI3K/Akt expression was increased as well. This prompted speculation that the PI3K/Akt pathway might be involved in the HO-1 regulation of Mfn1 expression. However, as knowledge is limited, the answer to this question is still unclear, and further investigation is still needed.

In mammalian cells, mitochondrial proteins Mfn1, Mfn2 and OPA1 are known as protein mediators of mitochondrial fusion. Previous studies suggested that Mfn1 was broadly expressed in different tissues and at high levels^30^. Lack of Mfn1 leads to fusion deficiency and results in severely fragmented mitochondria^31^. Furthermore, in Mfn1 knockout cells, more mitochondrial DNA (mtDNA) mutations were observed^32^. By contrast, overexpression of Mfn1 by transient transfection of cultured cells contributed to the formation of mitochondrial networks, whereas a point mutation in the Mfn1 gene resulted in dramatically attenuated mitochondrial fusion^31^. All these findings indicate that Mfn1 plays a key role in mitochondrial fusion machinery. Thus, in our research, the mRNA and protein expression of Mfn1 was measured to evaluate mitochondrial fusion changes. Similar to the current study, Hull TD *et al*. reported in 2016 that HO-1 has a novel role in protecting the heart from oxidative injury by regulating mitochondrial fusion proteins Mfn1 and Mfn2^33^. In that study, researchers treated WT mice with DOX, and the results suggested that the level of expression of Mfn1 and Mfn2 was strikingly elevated in cs-HO-1 mice after DOX treatment.

In conclusion, our study showed that the elevation of HO-1 expression could reduce oxidative stress in LPS-induced ALI/ARDS in rats. As our results suggested, this is the first report to reveal that HO-1 upregulation in *in vivo* and *in vitro* studies could play an indirect a role in mitochondrial fusion protein Mfn1 expression in LPS-induced ALI rats and alveolar macrophages. In addition, we further demonstrated that the possible mechanism might be due to PI3K/Akt pathway activation^34^,^35^, which has an effective impact on regulating HO-1 upregulation. Thus, our findings offer a new insight into the role of HO-1 in attenuating oxidative stress through the mitochondrial fusion protein Mfn1, which greatly contributes to further clarification of the mechanism of HO-1 protection against oxidative stress and provides opportunities for designing mitochondria-targeted HO-1 interventions for ALI/ARDS treatment.

## Materials and Methods

### *In vivo* study

#### Animals

Two-month-old healthy male Sprague-Dawley rats (160–180 g) were purchased from the Tianjin Institute of Acute Abdominal Diseases of Integrated Traditional Chinese and Western Medicine. The animal room was switched to dark after 12 h light each day. The rats were housed and given free access to a standard laboratory diet and drinking water. During the experiment, the environmental temperature was maintained between 23 °C and 25 °C. Approval was obtained from the Animal Use and Care Committee of Nan Kai Hospital, and the animals were managed in accordance with National Institutes of Health guidelines.

#### Experimental groups

Animals were divided into 10 groups (*n* = 10 each) as follows: Control (C), LPS (L), LPS + Hemin (LH), LPS + Znpp (LZ), LPS + Hemin + Znpp (LHZ), Hemin (H), Znpp (Z), Hemin + Znpp (HZ), LPS + NaHCO3 (LNaHCO3) and LPS + NaOH (LNaOH). Drugs were injected via the tail vein: C received 1 ml of 0.9% saline vehicle; LPS (LPS, Sigma, USA) was injected at a dose of 5 mg/kg dissolved in 1 ml of 0.9% saline vehicle; Hemin (Hemin, Sigma, USA) was injected at the dose of 100 mg/kg dissolved in 1 ml of NaOH; and zinc protoporphyrin-IX was injected at a dose of 10 μmol/kg dissolved in 1 ml of NaHCO_3_ (ZnPP-IX, Sigma, USA).

#### Sampling and storage

After 6 hours, the animals were sacrificed after intravenous injection of LPS or 0.9% saline vehicle. The lung tissues were immediately removed, and the samples were frozen in liquid nitrogen and stored at −70 °C until assayed. Rats that died during the 6 h were not included in the experimental results.

#### Measurement of MDA contents and SOD activity

The right upper lung was rinsed and weighed and then placed into tubes with 0.9% saline vehicle. Then, the tissue samples were homogenized for 10 minutes. After centrifugation at 3000 r/min for 10 minutes at 4 °C, MDA and SOD activity were assessed using an assay kit (Nanjing Jincheng Bioengineering Institute, Nanjing, China) according to the manufacturer’s instructions. One unit of SOD activity was defined as the amount of enzyme inhibiting NBT reduction by 50%. SOD activity was expressed as nU/mL culture media. MDA content was expressed as nmol/L culture media.

#### HE staining

The right middle lung from each rat was fixed in 10% formalin, embedded in paraffin, cut into 5 mm sections, and stained with H&E. We then evaluated the degree of injury in the lung tissue according to inflammatory cell infiltration in the airspace or vessel wall, alveolar congestion, hemorrhage, alveolar wall thickness and hyaline membrane formation.

#### Real-time reverse transcriptase-polymerase chain reaction (RT-PCR)

Total RNA was extracted from the right lower lung 6 h after LPS injection using Trizol (Invitrogen Life Technologies, Carlsbad, CA). Then, cDNA was created using the Transcriptor First Strand cDNA Synthesis Kit. RT-PCR was performed using SYBR green PCR master mix (Applied Biosystems, Foster City, CA) and a 7300 ABI-Prizm Sequence Detector (Applied Biosystems). Gene-specific primers were as follows: β-actin forward: 5′-GACAGGATGCAGAAGGAGATTACT-3′, reverse: 5′-TGATCCACATCTGCTGGAAGGT-3′ (142 bp); Mfn1 forward: 5′-TGGGGAGGTGCTGTCTCGGA-3′, reverse: 5′-ACCAATCCCGCTGGGGAGGA-3′ (118 bp); HO-1 forward: 5′-CGACAGCATGTCCCAGGATT-3′, reverse: 5′-TCGCTCTATCTCCTCTTCCAGG-3′ (160 bp). A 20 μl PCR reaction system was used, and the reaction conditions were as follows: 95 °C denaturation for 15 min, 95 °C denaturation for 10 s, 59 °C annealing extension for 30 s, for a total of 40 cycles. RT-PCR products were measured using a comparative CT method and 2^−ΔCT^ to represent the relative expression of the target gene. All of the assays were corrected by subtracting β-actin.

#### Western Blot Analysis

The protein expressions of HO-1 and Mfn1 of the left upper lung samples after 6 h were analyzed by Western blot. The proteins were extracted according to the instructions of the total protein and nuclear protein extraction kit (Thermo, USA), and the protein concentrations were detected using a BCA protein assay kit (Thermo, USA). Equal amounts of protein were fractionated using a 12% SDS-PAGE gel and were then transferred to a PVDF membrane (Millipore, USA). Blots were washed in triplicate for 5 min in TBS and were then incubated overnight at 4 °C with polyclonal rat antibodies against HO-1 (1:250, Abcam, UK) and Mfn1 (1:200, Santa, CA). Primary antibodies were diluted in a blocking solution containing 1% nonfat milk plus 0.5% BSA in TBS-0.05% Tween 20. After three washes with TBS-0.05% Tween 20, the blots were incubated at 37 °C for 1 h with horseradish peroxidase (HRP)-conjugated goat anti-rabbit IgG (1:3000 dilution, Beijing Kang-century Biotechnology Co, China). The blots were visualized with enhanced chemiluminesence (Bio-Rad, USA) according to the manufacturer’s instructions, and the relative densities of the bands were quantified by densitometry (Molecular Analyst Image-analysis Software, Bio-Rad, USA).

### *In vitro* study

#### Agents

The following materials were used in this study: Rat alveolar macrophage cells 8383 (ATCC, USA), LPS (Sigma, USA), Hemin (Sigma, USA) and ZnPP-IX (Sigma, USA). All chemicals were dissolved in dimethyl sulfoxide (DMSO), and the final concentration of DMSO in each treatment was less than 0.5%. HO-1, Mfn1, PI3K and pAkt antibodies were obtained from Santa Cruz Biotechnology (Santa Cruz, CA).

#### Cell culture and stimulation

Rats alveolar macrophage cells NR8383 were obtained from American Type Culture Collection (Manassas, VA). Cells were cultured in Dulbecco’s modified Eagle’s medium (DMEM; Gibco, Grand Island, NY) supplemented with 2 mM glutamine, antibiotics (100 U/mL of penicillin A and 100 U/mL of streptomycin), 10% heat-inactivated fetal bovine serum (Gibco/BRL), and they were maintained in a 37 °C humidified incubator containing 5% CO_2_. The adherent cells were subcultured with 0.25% trypsin digestion at a 1:4 scale after a single layer of cells were fused. A density of 4 × 10^4^/ml cells was treated in a 96-well culture plate with 200 μl/well and incubated for 24 h. Cells were treated with LPS at a dosage of 10 μg/ml. Then, cells were supplemented with 20 μM Hemin and (or) 10 μM ZnPP 1 h prior to LPS treatment.

#### Measurement of ROS and MDA concentrations and SOD activity

The level of ROS was detected by fluorescent probe dihydroethidium (DHE) (Molecular Probes) and carboxy-dichlorodihydrofluorescein (H2DCFDA) (Molecular Probes) according to the manufacturer’s instructions. Cells were loaded with 25 μm carboxy-H2DCFDA in PBS or 5 μm DHE in HEPES buffer at 37 °C for 30 minutes. Images were acquired at room temperature, and fluorescence intensity was measured using IPLab imaging software (Scanalytics, Inc.).

MDA concentration and SOD activity assays were performed as described in the *in vivo* study.

#### RNA extraction and PCR

RNA extraction and PCR were performed as described in the *in vivo* study.

#### Western blotting

Rats alveolar macrophage NR8383 cells were washed twice in ice-cold PBS and harvested by adding trypsin-EDTA (Sigma-Aldrich, St. Louis) to the culture dishes for 5–10 min. Trypsin activity was blocked using the trypsin inhibitor from glycine max (soya bean; Sigma-Aldrich, St. Louis) at a ratio of 10 mL per 1 cm^2^ of cells. Cells were lysed in lysis buffer (300 mM NaCl, 20 mM TRIS [pH 7.4], 1% Triton, 200 mM PMSF, 1 mM DTT, 2 mg/mL Leupeptin, 1 mg/mL Pepstatin). After centrifugation for 10 min at 12,000 *g*,cell supernatants were collected, and the protein concentrations of individual cell lysate samples were determined using a Bradford assay. A total of 25 mg of total protein was separated by SDS-PAGE and transferred to a nitrocellulose or PVDF membrane for HO-1, Mfn1, PI3K and pAkt measurement. Membranes were incubated with antibodies against HO-1, Mfn1, PI3K and pAkt (1/1000; Sigma-Aldrich). Actin (1/1000; Abcam, Cambridge, United Kingdom) was used as the control for the total and mitochondrial fractions. Membranes were incubated with the appropriate secondary antibodies conjugated to horseradish peroxidase (Santa Cruz Biotechnology). The blots were developed using the enhanced chemi-luminescence method. The band densities were analyzed using Image J software (NIH, Bethesda, MD, USA).

### Statistical Analysis

Statistical analysis was performed using GraphPad Prism 5 and SPSS 16.0 software. Data were expressed as the mean ± SEM, and statistical significance was determined using one-way analysis of variance (ANOVA) followed by Bonferroni’s correction. Statistical significance was considered at P < 0.05 or P < 0.01.

## Additional Information

**How to cite this article**: Yu, J. *et al*. Effect of Heme Oxygenase-1 on Mitofusin-1 protein in LPS-induced ALI/ARDS in rats. *Sci. Rep*. **6**, 36530; doi: 10.1038/srep36530 (2016).

**Publisher’s note:** Springer Nature remains neutral with regard to jurisdictional claims in published maps and institutional affiliations.

## Figures and Tables

**Table 1:** Oxidative stress measurements.

Parameters	Control	LPS	LPS + Hemin	LPS + ZnPP
ROS	110.562 ± 1.305	142.758 ± 4.444^a^	138.145 ± 3.919^ab^	148.544 ± 3.217^ab^
MDA	3.212 ± 0.388	4.5024 ± 0.518^a^	3.9016 ± 0.410^ab^	4.813 ± 0.472^ab^
SOD	70.586 ± 1.761	52.406 ± 1.519^a^	65.991 ± 1.839^ab^	47.305 ± 1.652^ab^

ROS and MDA production and SOD activity upon exposure to LPS conditions. In the control group(C), the levels of ROS and MDA production remained at low concentrations, whereas SOD activity remained at a high concentration (P < 0.05, Table 1). After LPS treatment, the levels of ROS and MDA contents were dramatically increased with decreased SOD activity in the LPS group(L) compared with the control group(C) (P < 0.05, Table 1). However, after Hemin pretreatment, our data showed that the levels of ROS and MDA contents were downregulated in the LPS + Hemin group(LH) compared with those of the LPS group(L). Concomitantly, SOD activity was upregulated (P < 0.05, Table 1). Moreover, we examined the levels of ROS and MDA contents and the SOD activity in the LPS + ZnPP-IX group(LZ). Our results suggested that treatment with ZnPP-IX increased the levels of ROS and MDA content, whereas it decreased the levels of SOD activity compared with those of the LPS group(L) (P < 0.05, Table 1).
